# What Should Be Considered When Assessing Hyperacusis? A Qualitative Analysis of Problems Reported by Hyperacusis Patients

**DOI:** 10.3390/brainsci12121615

**Published:** 2022-11-25

**Authors:** Kathryn Fackrell, Magdalena Sereda, Sandra Smith, Jacqueline Sheldrake, Derek James Hoare

**Affiliations:** 1 NIHR Nottingham Biomedical Research Centre, Ropewalk House, 113 The Ropewalk, Nottingham NG1 5DU, UK; 2 Hearing Sciences, Mental Health and Clinical Neurosciences, School of Medicine, University of Nottingham, Nottingham NG7 2UH, UK; 3 The Tinnitus and Hyperacusis Centre, London W1G 6JL, UK

**Keywords:** psycho-social, emotional, behavioural, clinical assessment, sub-typing hyperacusis

## Abstract

Hyperacusis (decreased sound tolerance) is a prevalent complaint. Yet, to date, no research has qualitatively evaluated the types of problems experienced by adults with hyperacusis. Our service evaluation aims to determine the hyperacusis-related problem domains reported by patients and the degree to which these domains were reported together. Retrospective analysis was conducted on an anonymised clinical dataset from 306 patients who attended a UK tinnitus and hyperacusis treatment centre between 1994 and 2017. Conventional content analysis was used to categorise responses to the question ‘Why is hyperacusis a problem?’ into domains which were then subjected to a cluster analysis. Twenty-five problem domains were identified, of which 12 were further classified into three overarching categories. ‘Fear’, ‘Reduced quality of life’ and ‘Physical reaction to sound’ were most frequently reported problems. Cluster analysis revealed that ‘Sleep difficulties’ and ‘Despondency’, were commonly reported together. Adults with hyperacusis face many challenges in their everyday lives. The nature of these problems indicates the need to develop complex interventions and assessments to aid management of hyperacusis. Current hyperacusis questionnaires may be useful in identifying some problem domains, but further assessment thorough patient interviews is required to fully explore all potential problems and make informed decisions about treatment.

## 1. Introduction

Hyperacusis is a hearing disorder characterised by “a reduced tolerance to sound(s) that are perceived as normal to the majority of the population or were perceived as normal to the person before their onset of hyperacusis, where “normal” refers to sounds that are generally well tolerated” [1] (p. 610). For the person experiencing hyperacusis, everyday sounds such as those produced by domestic appliances or traffic noise, can be uncomfortable or painful. The impact of hyperacusis on a person can vary from mildly problematic to incapacitating. The experience can result in significant distress, anxiety, and stress and a patient to have multiple negative reactions to a single sound [2,3,4]. People experiencing hyperacusis often avoid situations where noise levels are out of their control, and thereby become isolated and lose their independence; this not only affects the individual but also their family.

Current estimates suggest between 3.8% and 17.2% of adults in a general population experience hyperacusis, with the prevalence increasing with age [5]. Although potential mechanisms of hyperacusis such as 5-hydroxytryptamine (5-HT) dysfunction or auditory dysfunction have been proposed, it often remains medically unexplained, with no definitive diagnosis, aetiology, or cure [6]. A recent prioritisation exercise in the UK highlighted the need for research to focus on clinical needs and effective treatments, in particular the requirement for clearly defined guidelines on assessment and management of hyperacusis [7,8].

Reflecting the general lack of research, no standard clinical guidelines have been developed for hyperacusis. Treatment aims to help people manage their hyperacusis-related problems [9,10]. Decision making is therefore driven by what the clinician can offer, and what is indicated by assessment. Assessment relies on a variety of audiometric measures (e.g., uncomfortable loudness levels, most comfortable listening levels), patient self-report, and questionnaires [9,11]. Audiometric measures provide a measure of the “threshold of discomfort” for sound. However, these measures provide little information on the impact and negative consequences of hyperacusis [12,13,14]. Conversely, questionnaires can provide measures, to varying degrees, of different problems and consequences associated with hyperacusis. For example, the Hyperacusis Questionnaire (HQ) measures attentional deficits due to noise disturbance, social behavioural and emotional consequences of hyperacusis [15], the Inventory of Hyperacusis Symptoms (IHS) measures psychosocial impact, emotional arousal, functional impact, general loudness, and communication difficulties in relation to hyperacusis [16], the Multiple-Activity Scale for Hyperacusis (MASH) measures annoyance in relation to sound sensitivity [17], and the Hyperacusis Impact Questionnaire (HIQ) measures the impact of hyperacusis on a patient’s life [18].

Most hyperacusis questionnaires were developed with items and domains derived from analyses of previous questionnaires, clinical interview protocols, and clinical experience. The IHS was the only questionnaire to report that testimonies from patients contributed to item construction [16]. However, no further details were provided on what problems were specifically reported by the participants or how these testimonies contributed to the item construction and questionnaire structure (the domains/subscales). Furthermore, it has been suggested that hyperacusis could be divided into subtypes based on the distinctive reactions to sounds. In 2014, Tyler et al. [19] proposed that hyperacusis could be characterized into four broad subtypes of loudness, annoyance, fear, and pain, experienced either singly or in combination. However, a recent evaluation of 11 hyperacusis patients found that these subtypes do not necessarily discriminate different individuals [4]. Further work is therefore indicated to fully understand the different experiences of hyperacusis (what problems arise because of hyperacusis) and the potential subtypes of hyperacusis (the types of problems that are consistently reported together) and whether there are problems that people confuse or conflate with hyperacusis (e.g., difficulties with tinnitus or hearing loss). This is essential to informing clinical practice, service provision, and future research on hyperacusis.

Here, we report a service evaluation involving a retrospective analysis of anonymised data from 306 patients who attended a Tinnitus and Hyperacusis Centre in the United Kingdom. The aim was to determine the hyperacusis-related problems reported by people experiencing hyperacusis and examine which types of problems were consistently reported together. To our knowledge, this is the first qualitative evaluation to comprehensively do so.

## 2. Materials and Methods

This service evaluation involved a retrospective analysis of anonymised data that had been routinely collected from patients attending the Tinnitus and Hyperacusis Centre (London, UK) between 1997 and 2017. Ethical committee approval was not required to evaluate the service, and data use and analyses complied with the governance procedures of the data controller (J.S.).

### 2.1. Data Collection

The Tinnitus and Hyperacusis Initial Interview Form [20] was completed by an audiologist (J.S.) during the first consultation to assess each patient’s suitability for Tinnitus Retraining Therapy (TRT) [21]. The interview form consists of questions that evaluate tinnitus, sound tolerance (hyperacusis), hearing loss, including a 0–10 rating scale of how much each condition (tinnitus, hyperacusis, hearing loss) is a problem, where higher scores indicate larger problems. Questions about hyperacusis included the degree of severity, annoyance, and effect on life experienced over the last month (using a 0–10 rating scales), what percentage of time certain activities were prevented or affected by hyperacusis, and “Why is hyperacusis a problem?”. For this final question, verbatim responses were recorded on the interview form. The same questions were asked about tinnitus and/or hearing loss, if indicated.

### 2.2. Participants

The responses from 306 adult patients were included. Patients either attended the clinic with primary complaints of hyperacusis or complaints of both tinnitus and hyperacusis. Patients mean age was 45.2 years (SD = 14.1; Range = 14–81 years). One hundred and seventy-eight patients (58%) were men and 128 (42%) were women. Two-hundred and fifteen patients (82%) reported that they experienced physical discomfort due to their hyperacusis. Ninety-four (38%) reported the use of ear protection to reduce sound levels. Over 50% of patients reported going to concerts (n = 173) and social activities (n = 164) were affected or prevented 100% of the time. Whilst over 40% of patients reported going to the movies (n = 133) and doing housekeeping (n = 131), and over 30% of patients reported going to work (n = 98) and restaurants (n = 100), and childcare (n = 95), were affected or prevented 100% of the time.

All patients reported their hyperacusis was problematic, with an average of 5.5 (SD = 2.9, n = 306) on the 10-point problem scale, with 58% scoring more than 5. On ratings of severity, annoyance, and effect on life, patients averaged 6.4 (SD = 2.1, n = 291), 7.1 (SD = 2.5, n = 288) and 6.0 (SD = 2.7, n = 286) out of 10, respectively. The majority of patients scored more than 5 out of 10 for the degrees of severity (70%, n = 201), annoyance (75%, n = 215), and effect on life (60%, n = 170).

Of the 306 patients reporting problems, 274 (89%) also reported experiencing tinnitus and 107 (37%) also reported experiencing some hearing problems. Only 19 patients (7%) reported using hearing aids. When asked to rate how problematic their tinnitus and hearing loss was on the 10-point problem scale, patients averaged 5.6 (SD = 2.7, n = 270), and 3.0 (SD = 3.0, n = 193) out of 10, respectively.

### 2.3. Conventional Content Analysis

Data analysis of free-text responses was guided by the methodological framework for inductive content analysis [22,23]. This involved interpreting direct patient responses to develop new insights and understanding of hyperacusis-related problem, without imposing any pre-existing categories or theories. Because most responses to the question “Why is Hyperacusis a Problem?” were brief, they were coded using only what was written rather than what was implied. This avoided misinterpretation of meaning that could occur because of a lack of context [24]. Analyses were conducted by four researchers (KF, MS, SS, DH) experienced in this type of qualitative analysis.

Inductive content analysis involved (i) data immersion and familiarisation: read and re-read dataset; (ii) data reduction: create meaningful units/open codes (based on extracting exact words from data) and note any potential categories coming out of the data. Meaningful codes constituted one word, part of a sentence or a whole sentence that had to contain enough information to allow meaningful interpretation with respect to the research question; (iii) creating categories/data abstraction: compare similarities and differences in codes to identify and refine sub-categories (domains) and overarching categories [22,23,24]. Initially, two authors (KF, MS) independently immersed themselves in the whole dataset, before extracting meaningful problem codes from each response and noting potential categories. These authors examined and discussed their independently extracted codes and reached a consensus on the final problem codes. Disagreements were discussed until consensus was reached, involving other authors to reach a majority decision. Four authors (KF, MS, SS, DH) then reviewed all the problem codes and proposed initial sub-categories/domains. Any of the four authors could suggest a domain that they believed was representative of a subset of problem codes. These initial domains were further refined by four authors. During this process, proposed domains were checked for commonality or overlap of problem codes. Domains that conceptually represented a similar overarching concept were grouped together under an overarching category but kept as sub-domains within the category. For example, the domains of ‘Avoidance’ and ‘Protection’ were grouped under the overarching category of ‘Safety Behaviour’. This allowed for an overarching construct to be captured without losing the distinct elements of the domains. Initial domains with problem codes that conceptually overlapped were merged. For example, initial domains described as ‘Isolation’ and ‘Social interaction’ were combined to form ‘Reduced social interaction’. Initial domains with problem codes that through discussion were deemed to include more than one domain of problem were split to be distinct domains. This iterative process continued until every domain was deemed valid, with each code only being allocated to one domain [24]. As a final validation step, the audiologist (JS) who interviewed the patients reviewed the extracted problem codes and domain grouping, checking that the domains captured the essence of the raw data and represented the core themes from the data.

### 2.4. Hierarchical Cluster Analysis

A hierarchical cluster analysis was conducted to examine the degree to which identified domains were more consistently reported together within individual patient responses. Patients could technically report between one and 25 different problems. So, for example, patients reporting problem A (e.g., pain) also often report problem B (e.g., loudness). Data used for the analysis were a matrix of whether (score 1) or not (score 0) individual patients reported a problem within each of the 25 problem domains. Euclidean distance estimates were then plotted per patient in a theoretical 25-dimensional space (representing the 25 domains in our data). In this way, a shorter theoretical distance between points (domains) would indicate which domains of hyperacusis-related problems are most likely to group together, i.e., co-occur for an individual patient, or potentially that there was redundancy in the domains that have been identified, for example, domain A and domain B may be the same construct or overlap significantly. Hierarchical cluster analysis was conducted in PAST statistical analysis software version 4.06 [25,26].

## 3. Results

### 3.1. Conventional Content Analysis

From the 306 patient responses to the question “Why is hyperacusis a problem”, 443 meaningful problem codes were identified; 193 patients (63%) reported only one problem, 90 patients (29%) reported two problems, 22 (7%) patients reported three problems, and one patient reported four problems.

Following grouping and refining of the 443 meaningful codes, 25 problem domains were identified and agreed to cover the content of the raw data (Table 1). Of the 25 domains, 12 domains could be further grouped into overarching categories relating to ‘Feeling emotionally unsettled’ (8 domains), ‘Experiencing physical reactions to sounds’ (2 domains) and ‘Using safety behaviours’ (2 domains). Table 1 provides a description for each domain and overarching category, the pattern in frequency of reporting domains for full sample (n = 306), and for the hyperacusis-only sub-group (n = 32).

For all patients, the most frequently reported domains were ‘Fear’ (22%) and ‘Reduced quality of life’ (17%), whilst ‘Feeling “normal”’ and ‘Sleep difficulties had the fewest problem codes (3% and 2%, respectively). The ‘Emotionally unsettled’ category included eight domains that represented a range of emotional reactions associated with experiencing hyperacusis. Of those, ‘Fear’ was most often reported by patients as problematic (22%). This domain included patients reporting feeling fearful and worry about “*not knowing what the problem is*” or that their “*hearing is deteriorating”* or being *“frightened of making it worse”* or being “*afraid for the future*”. The ‘Reduced quality of life’ domain included problem codes that described hyperacusis as restricting and interfering with every aspect of life, with patients reporting that they “*can’t function with other sounds around*”, and that hyperacusis *“makes life difficult”* and *“takes a lot of pleasure from life*”. The remaining domains that were more frequently reported reflected the physical pain and discomfort caused by sounds (‘Experiencing physical reactions to sounds’) and the active behaviour that patients engaged in to avoid and protect themselves from sounds (‘Safety behaviour’) and the restrictions on activities that hyperacusis imposed (‘Activity restrictions’) (Table 1).

For patients only reporting hyperacusis (n = 32), the more frequently reported domains reflected those reported by the full sample. For this sub-group, ‘Reduced quality of life’ (31%) was most frequently reported by patients, followed by ‘Effects on relationships’ (19%), ‘Fear’ (16%), ‘Reduced social interactions’ (13%) and ‘Pain’ (13%). In terms of ‘Effects on relationships’, patients reported that hyperacusis “*affects all relationships*”, including “*family life*”, “*friends*” and partners, one patient reported being unable to “*sleep in the same bed as husband*”. Similarly, patients reported ‘Reduced social interactions’, whereby they felt that their hyperacusis “*influences what they [I] do socially*” and that they “*cannot go out with friends*” because of it. Eight problem domains were not reported by these patients, including ‘Difficulties concentrating and relaxing’, ‘Irritation’, or ‘Annoyance’ (Table 1).

### 3.2. Hierarchical Cluster Analysis

**Figure 1:**
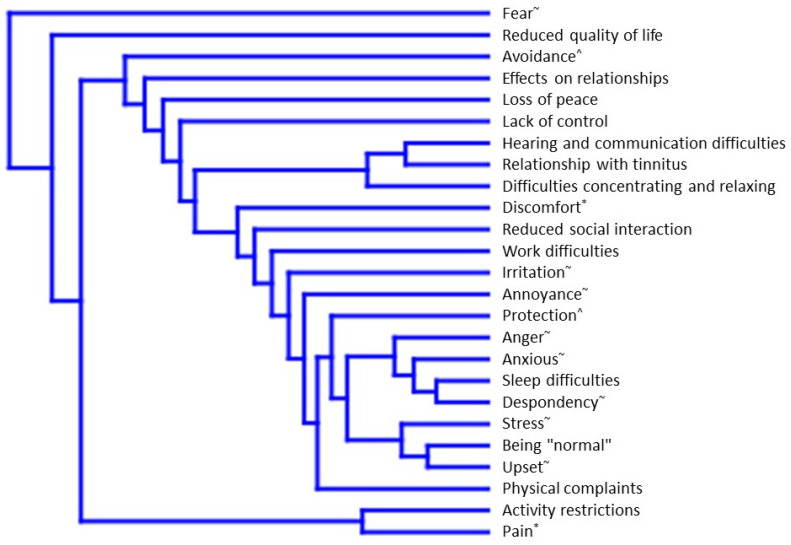
Cluster analysis indicating relatedness of hyperacusis problems within the responses from individual patients. * In overarching category: Experiencing physical reactions due to sound; ^ In overarching category: Using safety behaviour; ~ In overarching category: Feeling emotionally unsettled due to the experience of hyperacusis.

Cluster analysis suggested that the problem domains identified in this evaluation that co-occur are independent (Figure 1). The smaller the theoretical distance between domains, the more common it was for these domains to be reported together by the same patient. Hence, ‘Sleep difficulties’ and ‘Despondency’ most closely clustered (often reported together) and whilst these problems may be related, the results suggest that they are clearly different domains. Similarly, ‘Pain’ and ‘Activity restrictions’ clustered closely, which indicates that patients who experience pain may feel more restricted in what they can do because of it. Euclidean distances between domains when plotted in a 25-dimensional space are given in Supplementary Figure S1. ‘Fear’ and ‘Reduced quality of life’ were by far the most frequently reported by patients and hence in this analysis formed the outer clusters (i.e., they were more broadly present in this theoretical multi-dimensional space). Interestingly, the two domains ‘Hearing and communication difficulties’ and ‘Relationship to tinnitus’ clustered closely together, which may indicate some conflation of these problems. Furthermore, these hearing-related problems were next most closely clustered with ‘Difficulties concentrating and relaxing’ which again may point to conflation of hyperacusis-related problems with hearing loss- or tinnitus-related difficulties.

## 4. Discussion

This is the largest evaluation of its kind to date that has examined why hyperacusis is reported as a problem in a clinical population. Through our retrospective analysis of interview data from 306 patients attending a specialist tinnitus and hyperacusis clinic, we determined the types and patterns of problems that arise because of hyperacusis. Our analysis found 25 different problem domains associated with the experience of living with hyperacusis. Of these, ‘Fear’, ‘Reduced quality of life’, and ‘Pain’ were the most frequently reported problems.

### 4.1. Fear

‘Fear’ reflected patients’ fears of damaging their ears and hearing, fears of making their hyperacusis worse, and fears for their future with hyperacusis. Within the diagnostic categories proposed by Tyler et al. [19], ‘Fear hyperacusis’ was defined as an aversive response to sounds, either a particular sound or a class of sounds that results in anticipatory and avoidance behaviour, including people taking steps to reduce participation in activities and avoiding situations where they fear these sounds might occur. Our data show that although there was an aversive response that focused on fear of damage or making hyperacusis worse, this reaction was not aligned with fear of specific sounds. Rather, patients did not specify particular sounds or a class of sounds, and fear was described in relation to all sounds and situations. Similarly, an investigation into the relationship between fear and hyperacusis in William’s syndrome concluded that irrespective of sound or noise incidence, hyperacusis could be connected to both fear and anxiety and that this fear could develop into more generalised anxiety and phobias [27]. It is also possible that the fear being experienced may be due to the overactivation of a hypervigilance network within the brain [28,29]. It has been proposed that increased activation of the anterior cingulate and orbitofrontal cortex may lead to constant increased vigilance and alertness to sounds and through this hypervigilance an increased feeling of fear could occur. Furthermore, a pre-existing increased activation within this network or pre-existing feelings of fear may have led to the emergence of hyperacusis [29].

Fear or feelings of being afraid, occurs when there is awareness, through cognitive processing of the situation, that personal well-being is being challenged or threatened [30]. In this case, everyday sounds challenge physical, mental and social well-being, to name some. In this evaluation, fear was often reported alongside the other problems domains, suggesting that fear is a dominant characteristic of hyperacusis. Meeus et al. [13] also found that patients who were afraid of sound scored significantly higher on the MASH, the HQ global score, and the HQ attentional and social subscales, than patients who did not report being afraid of sounds. Furthermore, Tyler et al. [19] concluded that fear hyperacusis could be experienced alone or in combination with the other three categories they described. Our findings suggest that the latter is more likely. Fear could therefore play a key role maintaining and exacerbating the other problems identified here, such as activity restrictions, reduced social interaction and safety behaviours. The latter, for example, can occur in the face of a perceived threat or in anticipation of a perceived threat and feeling fearful [31,32]. Our findings would suggest that when patients feel threatened or afraid/fearful due to the sounds or the anticipation of sounds, they might use ‘direct avoidance’ of situations or ear protection within the situation (e.g., ‘subtle avoidance’) to reduce fear [33]. Due to this, many patients might resist changing or discarding their safety behaviours, and therefore careful use of safety behaviours in the early stages of treatment may be warranted especially in patients exhibiting heightened fear [32].

Tyler el al.’s [19] definition of ‘Fear hyperacusis’ is often considered as equivalent to phonophobia, which involves a persistent, dominant fearful reaction to specific sounds, irrespective of the intensity of the sound [34,35]. However, Jastreboff and Jastreboff [36,37] suggested that phonophobia is a sub-category of misophonia (‘an abnormally strong reaction to a sound with a specific pattern and/or meaning to an individual’), and that hyperacusis and misophonia are two frequently coexisting components of decreased sound tolerance (‘negative reactions following exposure to sound that would not evoke the same response in an average listener’). Our findings challenge how distinctive phonophobia is from hyperacusis and raises the question whether it should be considered a specific sub-category of hyperacusis such as that proposed by Tyler et al. [19].

In terms of measuring fear in hyperacusis, some consideration has been given to fear within the HQ [15], the German Questionnaire on Hypersensitivity to Sound (Geräuschüberempfindlichkeitsfragebogen—GÜF) [38], and IHS [16]. Despite being developed prior to the emphasis on fear within the diagnostic framework for hyperacusis [19], the HQ [15] does include one item that the authors proposed measured fear of noise (item 9). The GÜF [38] also includes three items that focus on being afraid of sounds and noise (Items 1 and 6) and being afraid that loud sounds might damage hearing (item 14) [39]. Conversely, the development of the IHS [16], in particular the item selection and refinement, was based on the four hyperacusis categories proposed by Tyler et al. [19]. This resulted in just two items measuring how afraid patients feel in general because of sound sensitivity (item 16) and fear of being exposed to loud sounds on leaving the home (item 22). Therefore, hyperacusis questionnaires do provide some measure or indication of fear in relation to sound, however as yet there is no composite measure of fear in hyperacusis or subscale within a questionnaire that primarily focuses on fear that can be used to evaluate the effectiveness of management strategies in decreasing fear.

### 4.2. Reduced Quality of Life

‘Reduced quality of life’ was commonly reported. This domain was conceptualised as a general deterioration and interference in every aspect of daily life. Patients often referred to the difficulties hyperacusis caused to daily life as a whole and that hyperacusis took any enjoyment and pleasure out of life. In contrast to the ‘reduced quality of life’ domain identified for tinnitus by Watts et al. [40] with a related dataset, reported problems of ‘difficulties with work’ and ‘reduced social life’ were differentiated from this general reduced quality of life. Quality of life is often referenced in the literature in relation to the impact of hyperacusis, but there has been limited research investigating the relationship between hyperacusis and general quality of life and very few measures of hyperacusis-specific health-related quality of life currently exist. For those that do exist, quality of life is considered an over-arching concept, not a distinct concept of hyperacusis-related problems that would require specific items or subscales to measure it. For example, the IHS [16] was designed to measure the multiple dimensions of quality of life, mental health, and general functioning in relation to hyperacusis. This aligns with our conceptualisation of ‘Reduced quality of life’ as covering the general impact of hyperacusis on daily lives and, as such, although quality of life is important, it is not necessarily a problem domain that can indicate the type of treatment or management options needed.

### 4.3. Pain

‘Pain’ was a frequently reported problem by patients as a consequence of sounds. The description of the nature of pain was variable across patients with some making no reference to nature of the pain and just referring to the problem with sound as being “*really painful*”, whilst others used words such as “*piercing*” or “*sharp*” to describe the experience. This reflects Tyler et al.’s [19] description of pain hyperacusis as “the experience of pain at much lower sound levels than listeners with normal hearing (typically around 120 dB SPL)” (p. 404). In 2019, a survey conducted by Hyperacusis Research Limited with 350 people experiencing hyperacusis with or without tinnitus reported that 92% of respondents reported sounds causing pain or physical discomfort [41]. Although pain was not as large a problem in our population, with 70% of patients reporting physical discomfort, it does appear to be an important consideration in research and clinical assessments/management. Sensitivity to sounds and pain perceptions can be accompanied by psychological factors, such as stress and anxiety, as reflected in the problem domains identified in this evaluation [42,43]. Therefore, psychological factors should be considered during assessment and management as potential mediators of hyperacusis-related pain. As yet, measuring the pain experienced because of sound has not really been fully explored. Although Tyler and colleagues proposed the diagnostic categories for hyperacusis, measuring pain hyperacusis was not discussed [10,19]. The GÜF and IHS only include one question asking about sounds causing pain [16,38,39]. Single item measures of pain have been shown to be beneficial and informative when assessing chronic pain [44]. However, a single item will not capture the complexity of pain symptomatology [44,45] or reliably measure therapeutic changes in perceived pain following an intervention [45,46]. Therefore, there is a need to develop a self-report questionnaire measure of pain in hyperacusis.

‘Activity restrictions’ was frequently reported alongside ‘Pain’. This included patients reporting that hyperacusis “*stops me doing stuff I normally do*” and “*prevents me from doing things I enjoy*”. It is not unusual for people experiencing pain to alter or restrict their activities [47], and activity restrictions can be strategic, natural, and a responsible response to pain [48]. It is logical that people who experience pain because of sound feel they are restricted in what they can do because of the pain and because sounds are always present, and this will have implications for clinical management.

### 4.4. Loudness

Loudness is often considered as a primary psychoacoustic response and is considered a sub-category of hyperacusis [19]. The 2019 survey by the Hyperacusis Research Limited reported that ‘pain caused by sound’, ‘loudness sensation’ and ‘increased tinnitus from sound’ were most frequently endorsed as a primary complaint of hyperacusis [41]. Although, two of the primary complaints are reflected in our findings, it is perhaps surprising that loudness was not reported as a distinct problem within our patient population. Only two patients refer to loudness within our evaluation, one in relation to being “*afraid of loud sounds*” and the other in relation to the pain being experienced when “*hearing sharp loud sounds*”. The lack of reported problems with loudness may reflect the population or that loudness is not as distinct a problem as previous assumed. In their comparison of pain and loudness hyperacusis phenotypes, Williams et al. [35] found most tested variables were equivalent across the pain and loudness hyperacusis sub-groups. Although pain hyperacusis was potentially a more severe experience than loudness, both groups reported experiencing pain and loudness, temporary exacerbations of symptoms in response to sound exposure (setbacks), burden of comorbid conditions including tinnitus and headache disorders, functional impairment (quality of life) and the experience of pain. For example, both groups commonly endorsed experiencing intermittent pain (stabbing and sharp pain) and less frequently endorsed experiencing continuous pain (throbbing and dull). Therefore, it might be hard to meaningfully differentiate the experience of pain from loudness and vice versa, and clinicians and researcher alike should take this into consideration when assessing hyperacusis and evaluating interventions. Future research should explore these proposed sub-categories of hyperacusis to understand the interaction between the two and whether they can ever truly be distinct.

### 4.5. Hyperacusis and Tinnitus

‘Hearing and communication difficulties’ and ‘Relationship to tinnitus’ were most frequently reported together. Hearing difficulties are often associated with experiences of tinnitus [40]. Patients commonly struggle to disentangle hearing difficulties related to tinnitus from those related to hearing loss [49,50]. In this evaluation, 89% of patients reported tinnitus as well as hyperacusis. Therefore, that these domains were commonly reported together may reflect conflation of the two problems. However, our hyperacusis-only sub-group also reported ‘Hearing and communication difficulties’, especially hearing in loud noise situations. Vielsmeier et al. [51] found that when tinnitus severity was controlled for, hyperacusis was significantly associated with speech comprehension difficulties in noisy environments, but not in quiet environments. The more severe hyperacusis was, the more speech comprehension was a problem in noisy environments. Therefore, ‘Hearing and communication difficulties’ for hyperacusis might relate to either the level of noise in the environment or the tinnitus/hearing loss being experienced. Thus, a full clinical evaluation is indicated to explore these problem domains with the patient and remove any uncertainty around the precise hearing difficulties being experienced due to their hyperacusis. Another domain which might be related to the experience of living with both tinnitus and hyperacusis was ‘Difficulties concentrating and relaxing’. This domain was often reported alongside ‘Relationship to tinnitus’ and ‘Hearing and communication difficulties’. None of the patients in the hyperacusis-only sub-group reported ‘Difficulties concentrating and relaxing’. In a previous evaluation identifying problem domains in a tinnitus population [40], ‘inability to concentrate’ was one of the most frequently reported problems by patients, whilst ‘inability to relax’ was incorporated into ‘reduced quality of life’ which was the most frequently reported problem. A recent systematic review on tinnitus and cognition concluded that tinnitus is associated with poor performance across a range of cognitive domains, including executive functioning, processing speed and short-term memory [52]. In contrast, although in hyperacusis difficulties concentrating have been described in relation to anticipation of loud sounds or being in noisy environments [19,53], the relationship between hyperacusis and concentration is less well understood or researched. Further research is needed to fully understand the impact of hyperacusis on concentration/cognition and relaxation. Finally, the two most frequently reported domains in this evaluation (‘Fear’ and ‘Reduced quality of life’) were also the most frequently reported domains for a tinnitus population [40]. This indicates that ‘Fear’ may be a systemic problem for both of these complaints and that there is a need for comprehensive, reliable and clear information early in the patient pathway. Early treatment or support is critical to improving sleep, anxieties and reducing fears from growing in the first place [54]. Although there have been many advancements in tinnitus research including the development of the fear-avoidance model for tinnitus [55,56] and the availability of reliable information and support for patients with tinnitus [57], the same cannot be said for hyperacusis research and information. In a recent evaluation of information provided online for hyperacusis, Smith et al. [58] found that there was wide disparity in the quality and content of hyperacusis information across websites, with little or poor quality information provided on causes, onset and treatments. Therefore, future research should focus on untangling the problems associated with tinnitus from those associated with hyperacusis and developing high quality and reliable informational resources that provide guidance on seeking care and support.

### 4.6. Strengths and Limitations

One strength of the current evaluation was the availability of a large sample of clinical data on hyperacusis-related problems collected in a consistent manner over many years. Another strength of the evaluation was the rigorous nature of the qualitative analytical process undertaken. To reduce the potential bias associated with the subjective nature of qualitative research, our four experienced analysts used a standardised process to identify, define, and agree on the domains, and a final consultation with an experienced clinician to ensure that the domains identified captured the essence of the raw data.

There are limitations of this evaluation to consider. Firstly, our sample included patients reporting tinnitus as well as hyperacusis, with 89% of patients reporting both. Given patients were attending a specialised tinnitus and hyperacusis clinic, and tinnitus is commonly comorbid with hyperacusis, it is not unexpected that many patients would experience both [2,35]. It does however mean that some problem domains identified in this evaluation may represent problems associated with experiencing hyperacusis and tinnitus together. Secondly, the problem domains were based on responses from patients attending a private clinic. This would indicate a more affluent socio-economic group may be represented here than had this service evaluation been completed through a publicly funded clinic.

### 4.7. Clinical Implications

Current management strategies for hyperacusis include counselling, cognitive behavioural therapy, tinnitus retraining therapy and sound therapy management which are aimed at addressing both the physical-based components of hyperacusis and the psychological problems associated with the experience of hyperacusis [9,10]. For example, collaborative counselling may help reduce feelings of fear or avoidance through encouraging patients’ to express their fear of being exposed to sound and providing coping mechanisms to manage these difficult situations whilst sound therapy may help alleviate hyperacusis through gradual exposure to sounds (level and/or duration) and positive reinforcement [9,10]. However, there is a lack of sufficient evidence on the effectiveness of these management strategies for hyperacusis [9]. It is feasible to assume that the different problems reported here may require different management strategies. For example, transdiagnostic interventions that aim to address all the processes important for therapeutic progress (e.g., focusing on shared cognitive-emotional factors between pain and hyperacusis) and can be tailored to the individual needs of the patients [59]. Furthermore, sound therapy may not be as effective or beneficial for alleviating pain as it is for loudness, and this was not found to be as problematic for our patients [35]. As yet, there are no management strategies with a specific focus on decreasing hyperacusis-related fear or pain. One viable option would be to consider adapting the fear-avoidance model for pain [60] which offers a cognitive-behavioural explanation of why some patients struggle with the experience of ongoing pain and come to fear pain. Therapy based on this model has been shown to be effective in reducing fear of pain in chronic pain [61,62,63] and in 2011 was successfully adapted for tinnitus [56,64]. A recent James Lind Alliance prioritisation exercise for hyperacusis highlighted the need for research to address key priority areas, including identifying effective management strategies for hyperacusis and effective approaches for the different types or severities of hyperacusis [7,8].

## 5. Conclusions

The 25 problem domains identified in this service evaluation provide a major step to understanding the diverse experience of hyperacusis and provide an indication of the type of problems that should be considered in the assessment and management of hyperacusis including measuring treatment-related changes. Some of the existing hyperacusis questionnaires could be to probe some of the problems reported here, however development of questionnaires that provide multi-item measures of fear and pain in relation to hyperacusis are warranted. Whilst it may not be feasible to have a questionnaire that has subscales for each of the 25 domains; for responsiveness each domain would require at least three items to be included [65], which would then result in a 75-item questionnaire. This would not be practical for use in every clinical or research situation. Furthermore, it is unlikely that all these domains would need to be covered in every clinical assessment. One possible action is to identify the relevant domains to measure at the clinical assessment and remove any irrelevant domains for follow-up assessment. Another possible action is to consider grouping domains that are theoretically related together into scales. For example, safety behaviours and fear are theoretically linked and could possibly be grouped into one scale. Another action would be to gain consensus on the most important domains to measure for hyperacusis from all key stakeholders (patients, clinicians and researchers) and from this create a set of core domains that should be measured in clinical trials of interventions for hyperacusis. Future research needs to continue to advance our understanding of the experiences and impact of hyperacusis, whether these problems are replicated in other populations and to identify the best management strategies, assessment tools, and support to offer.

## Figures and Tables

**Table 1:** The 25 domains for hyperacusis problems and example quotes.

Overarching Category	Domain		% (n) of Codes per Problem	
	Description of Domain	*Example Quotes*	Full ^1^	Hy only ^2^
**Feeling emotionally unsettled due to the experience of hyperacusis**	**Fear** Feeling fearful, frightened and worried that something is wrong, of making it worse and for the future.	*“Afraid hearing is deteriorating”; Frightened of damaging ears more”; “Fearful of making it worse”; “Because you don’t know what’s coming.”*	22% (67)	16% (5)
	**Irritation** Feeling generally irritated because of intrusive sounds	*“Everyday things can drive me mad”; “It’s an irritation”; “Intrusive sound = irritation”*	4% (11)	—
	**Annoyance** Feeling annoyed because of sensitivity to sounds	*“It annoys me greatly”; “Annoyance because it shocks me”; “some noises that annoys me”*	3% (10)	—
	**Upset** Upsetting and distressing to experience hyperacusis	*“it distresses me”; “it’s upsetting, represents what I am upset with”; “Distressing in loud”*	2% (7)	—
	**Anger** Feeling angry and reactive	*“makes me want to scream”; “it makes me so angry”; “my reaction is out of order”*	2% (7)	6% (2)
	**Stress** Feeling stressed about the experience	*“because my hearing has always been so good its stressful to know that I’m hearing wrong; “I’m stressed all the time”*	2% (7)	3% (1)
	**Despondency** Feeling down, low and despondent	*“getting me down”; “I feel low about leaving”*	2% (6)	—
	**Anxious** Feeling anxious	*“makes me more anxious; “anxious about noise exposure”*	2% (6)	6% (2)
**Experiencing physical reactions due to sound**	**Pain** Feeling physical pain due to sounds	*“gives me pain”; “goes through head”*	10% (31)	13% (4)
	**Discomfort** Feeling physical discomfort due to sounds	*“The sounds everywhere are uncomfortable.”; “creates unpleasant feelings in my ears”*	5% (16)	3% (1)
**Using safety behaviour**	**Avoidance** Patients engage in active safety behaviour using avoidance to cope with situations	*“having to avoid situations (which becomes like jigsaw puzzle)”; “avoids doing things in certain way”*	8% (26)	3% (1)
	**Protection** Patients engage in active safety behaviour using protection to cope with situations	*“I have to cover my ears”; “i don’t feel i can play without my plugs”*	3% (10)	3% (1)
	**Reduced quality of life** Hyperacusis restricts and impacts on every element of daily life	*“It totally restricts me”; “Overall I’m not living, I’m surviving”; “destroys everyday enjoyment of life”*	17% (51)	31% (10)
	**Activity Restrictions** Hyperacusis imposes restrictions on activities.	*“I can’t do what I planned”; “I can’t listen to music”; “it stops me doing stuff I normally do”*	9% (28)	9% (3)
	**Effects on relationships** Hyperacusis effects all relationships including others adapting their behaviour to accommodate and lacking understanding	*“Other people don’t understand what is going on with me”; “when I am with family, they have to adapt for me”; “I can’t take the kids out or interact properly”*	8% (23)	19% (6)
	**Loss of peace** A loss of peace and quiet due to awareness and intrusiveness of sound	*“continued intrusion”; “odd to be aware of everyday noise”*	7% (22)	3% (1)
	**Lack of control** Feeling a lack of control and being unable to overcome it	*“unable to control and manage the sounds coming at me”; “it’s beginning to control me”*	6% (17)	9% (3)
	**Hearing & communication difficulties** Hyperacusis disrupts hearing, distorts sounds and makes communication difficult	*“distorts on phone”; “it makes it difficult to communicate normally”; “decreasing hearing”*	6% (17)	6% (2)
	**Reduced social interaction** Hyperacusis leads to feelings of isolation and being unable to socialise	*“feel isolated sometimes”; “stops social life almost completely”*	5% (15)	13% (4)
	**Work difficulties** Hyperacusis interferes and prevents work and career progression	*“Interferes with daily work.”; “Potential threat to my work”; “It’s stopping me be who I’m meant to be-a musician”*	5% (15)	9% (3)
	**Relationship with tinnitus** Belief that hyperacusis causes tinnitus and/or can make tinnitus worse	*“I think it might trigger the tinnitus”; “ makes tinnitus worse”*	5% (15)	—
	**Difficulties concentrating & relaxing** Hyperacusis is perceived as distracting, making it hard to concentrate and relax	*“it affects what I can concentrate on”; “can’t totally relax”*	4% (13)	—
	**Physical complaints** Other physical complaints associated with hyperacusis	*“gives me headache”; “makes epilepsy worse”*	3% (10)	9% (3)
	**Feeling “normal”** Feeling different about sound than others or how it was before	*“stops me feeling normal”; “It’s different from how it was”*	3% (8)	—
	**Sleep difficulties** Hyperacusis disturbs and interferes with sleep	*“can’t sleep if there are sounds around”; “interferes with sleep”*	2% (5)	—

^1^ Full = Full sample = n306. ^2^ Hy only = Hyperacusis sub-group = n36. — = no codes.

## Data Availability

The complete set of codes underpinning the current service evaluation is available from the authors.

## Supplementary Materials

The following supporting information can be downloaded at: https://www.mdpi.com/article/10.3390/brainsci12121615/s1, Figure S1: Euclidian distances plotted in a 25-dimensional space.
