# Supplementary figures and images for: What Should Be Considered When Assessing Hyperacusis? A Qualitative Analysis of Problems Reported by Hyperacusis Patients
Source: Brain Sci. 2022 Nov 25;12(12):1615. doi: 10.3390/brainsci12121615 (PMC9775019; doi:10.3390/brainsci12121615)

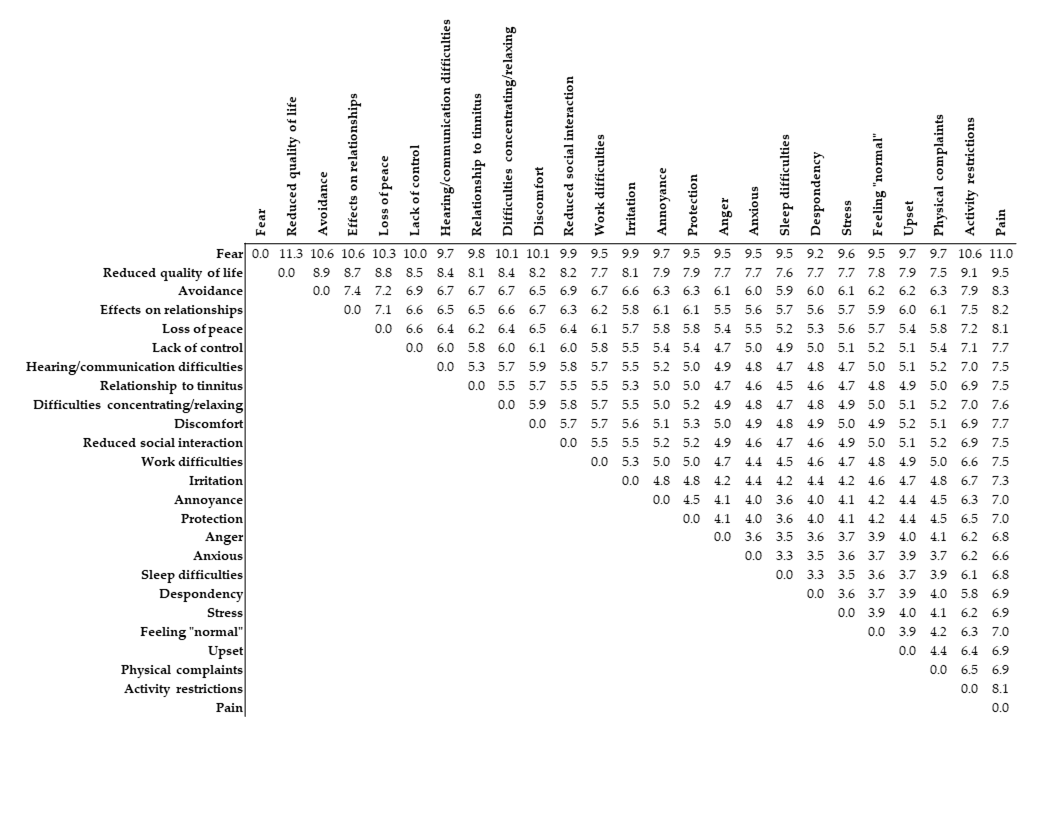

Supplement: Supplementary file 1 [file brainsci-12-01615-s001.zip › Supplementary Figure S1. Euclidian distances plotted.tif]
